# Primary renal mucormycosis in a type 2 diabetic patient: A case report from Syria

**DOI:** 10.1016/j.heliyon.2024.e32906

**Published:** 2024-06-15

**Authors:** Ali Jawad, Zein Alabdin Hannouneh, Hadi Salame, Hussein Taher, Banan Alkharat

**Affiliations:** a Faculty of Medicine, Damascus university, Damascus, Syrian Arab Republic; b Faculty of Medicine, Al Andalus University for Medical Sciences, Tartus, Syrian Arab Republic; c Faculty of Medicine, Al-Sham Private University, Damascus, Syrian Arab Republic; d Department of Infectious Diseases, Damascus University Hospital, Damascus, Syrian Arab Republic

**Keywords:** Mucormycosis, Zygomycosis, Renal mucormycosis, Diabetic ketoacidosis, Bronchopleural fistula

## Abstract

Mucormycosis is an opportunistic fungal infection that primarily affects immunocompromised individuals and rarely presents as renal mucormycosis (RM). Diagnosis can be challenging for many physicians. We report a rare case of primary, unilateral RM which triggered diabetic ketoacidosis in a type 2 diabetic patient. The case was later complicated by a bronchopleural fistula and a meropenem-resistant *Klebsiella* infection. The patient was ultimately treated with surgical intervention, liposomal amphotericin B, and polymyxine E. Early diagnosis and timely treatment of this life-threatening disease and its complications are significant in reducing mortality.

## Graphical abstract

**Figure:**
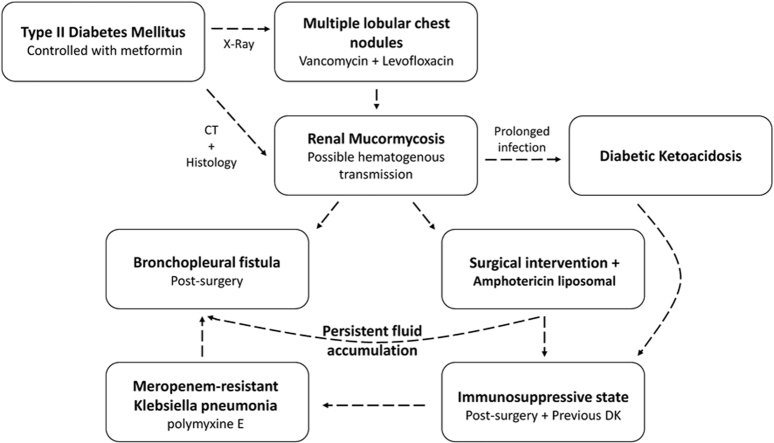

## 1 Introduction

Mucormycosis is a rare opportunistic fungal infection that predominantly affects immunocompromised individuals. Susceptible individuals include patients with uncontrolled diabetes mellitus, organ transplants, malignancies, malnourishment, major trauma, and intravenous drug abuse [1]. Renal mucormycosis (RM) is an unusual manifestation of invasive mucormycosis that is associated with a significant mortality rate [2]. The presentation of mucormycosis is diverse, and the clinical manifestations are influenced by both route of entry and predisposing conditions [3]. Typically, RM exhibits a rapid and aggressive progression rate and can manifest with fever, flank pain, and acute renal failure [2]. Standard treatment typically involves the administration of liposomal amphotericin B (LAmB) in conjunction with surgical intervention when feasible [4]. This report demonstrates an exceedingly rare case of primary RM in a type-2 diabetic patient, presenting with loss of consciousness and diabetic ketoacidosis (DKA).

### 1.1 Case presentation

A 30-year-old, incarcerated male presented to the emergency department with loss of consciousness. He is a smoker with controlled diabetes mellitus type 2 (DM-2) on metformin. Upon arrival, the patient exhibited pyrexia and was in shock. His vitals included blood pressure of 80/50 mmHg, respiratory rate of 40 breaths per minute, heart rate of 130 beats/minute, oxygen saturation of 82 %, and temperature of 38.5 ֯C. Laboratory values revealed DKA with mild elevation of ALT and AST and an increase in ESR, CRP, and ferritin.

Subsequent to a chest x-ray revealing multiple lobular nodules, the patient was started on vancomycin (1g x 2) and levofloxacin (750 mg). Two days later, the patient was alert and oriented. He restored most of his vitals except for his persistent fever, and he complained of acute flank pain. Lab investigations were insignificant except for a white blood cell (WBC) count of 17,000. Urinalysis displayed 50 WBCs. Urine and blood cultures were negative.

**Fig. 1:**
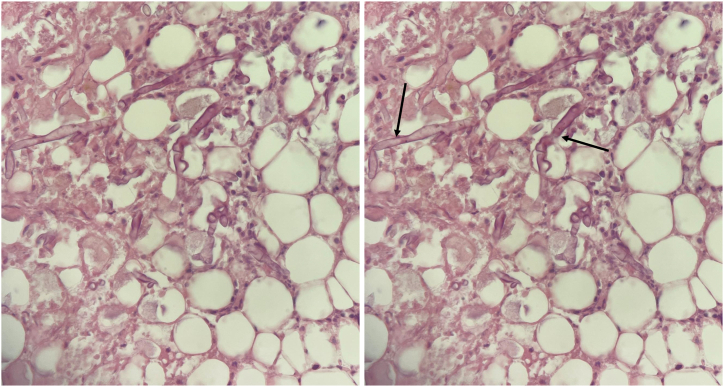
Periodic Acid Schiff (PAS) stain displaying mucormycosis with broad fungal hyphae (arrows) branching at 90° (magnification 400x).

An abdominal multi-slice computed tomography (CT) scan revealed bilateral, mild pleural effusion and an enlarged, destroyed right kidney. The parenchymal gas accumulation and surrounding edema was consistent with emphysematous pyelonephritis. The left kidney was hypertrophic. Other findings were unremarkable, except for a 2.5 × 3.5 cm fluid collection within the liver. The patient underwent urgent surgery to excise the affected kidney and adjunctive thoracentesis to drain the pleural effusion. Histopathological examination (HE) identified necrosis throughout renal tissue and perirenal adipose tissue, along with fungal thrombi in the hilar blood vessels [Fig. 1]. No neoplastic changes were observed. A diagnosis of extensive angioinvasive mucormycosis was established. Pleural fluid consisted of non-purulent exudate effusion with negative cultures and gram staining. The patient was started on LAmB (5 mg/kg, 7 flacons per day). Additionally, the patient was put on intensive glycemic control for 20 days. The patient's symptoms significantly improved post-operatively, and, within two months, he was able to carry out his personal activities.

**Fig. 2:**
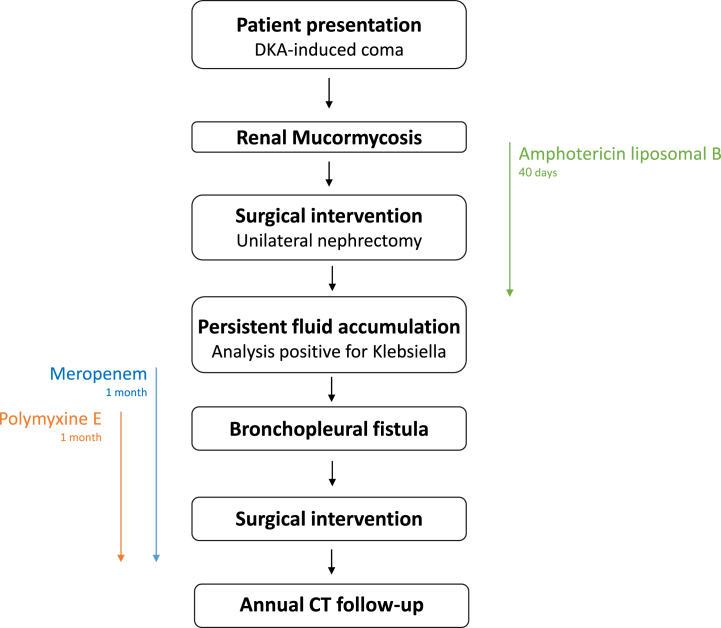
Patient events timeline

A follow-up CT scan demonstrated an increase in fluid collection within the liver and dense fluid accumulation in the right renal space, extending towards the sixth piece of the right liver lobe. The fluid was drained percutaneously, and fluid analysis revealed a positive culture for extended-spectrum β-lactamase-producing *Klebsiella*. A 14-day course of meropenem (1g x3) was initiated accordingly [Fig. 2].

A subsequent CT showed no improvement. The presence of a fistula, potentially originating from either the intestines or the chest, was suspected. Another CT scan with contrast demonstrated a bronchopleural fistula, which required surgical intervention. Pus collected during the surgical procedure revealed *Klebsiella* harboring resistance to meropenem. The patient was started on polymyxine E (2 million IU x3). Significant improvement was observed post-operatively with no recurrences or abscesses within a year of follow-up, and an annual CT was scheduled.

## 2 Discussion

Mucormycosis is a group of rare, fungal invasive infections characterized by a high mortality rate and caused by various species of *Mucorales* [2]. Invasive mucormycosis is categorized into six distinct clinical syndromes: rhino-orbital cerebral, pulmonary, gastrointestinal, cutaneous, disseminated, and miscellaneous [1]. The disseminated form carries worse prognosis and is characterized by hematogenous spread to other organs, most commonly, the brain [5]. Renal involvement is a rare presentation, occurring in 20–22 % of disseminated mucormycosis [2,6]. Primary RM is an extremely uncommon manifestation of mucormycosis with few documented cases in the literature [7]. The cause of isolated involvement may be due to hematogenous transmission, originating from an asymptomatic subclinical pulmonary source, similar to tuberculosis affecting the kidneys. Retrograde spread from lower urinary tract infection (UTI) is another plausible cause [2]. *Mucorales* can lead to life-threatening, widespread infections in individuals with compromised immunity, including those with DKA, hematologic malignancies, solid cancers, severe malnutrition, deferoxamine chelation, and other debilitating conditions [1,3]. In our report, the patient's diabetes was controlled with metformin, which reduces susceptibility. Additionally, we believe that DKA, which is an uncommon complication of DM-2, developed as a result of disseminated long-term infection in this case, rather than DKA being a predisposing factor for mucormycosis. The patient's primary renal presentation in addition to DKA is extremely rare. Nevertheless, his pneumonia suggests the potential subclinical hematogenous spread from the lungs.

The symptoms of RM can vary. According to Gupta et al. [7], the presentation of RM can include fever, flank pain and tenderness, pyuria and hematuria, and co-existing bacterial UTI. Acute renal failure was observed in 92 % of patients with bilateral kidney involvement [8]. Our patient initially presented with DKA, fever, and acute flank pain.

Ultrasonography and contrast-enhanced CT can aid in early diagnosis of RM [9]. Enlarged kidneys, hypodensities, and perinephric stranding are typical characteristics seen on contrast-enhanced CT scans [9]. However, the definitive diagnosis of mucormycosis is typically confirmed through HE, which demonstrates broad aseptate hyphae branching at wide angles [2]. Molecular diagnosis using real-time PCR has been recently suggested as a potential tool for early detection of this condition [10]. Our case demonstrated CT characteristics of unilateral emphysematous pyelonephritis. RM was later confirmed through HE [Fig. 1]. However, mucormycosis was not isolated in the lungs despite the suggested pulmonary source, which poses a limitation to this case. Imaging also demonstrated mild, bilateral pleural effusion and a hepatic fluid collection.

Key elements of successful therapy include initiating antifungal treatment promptly through early diagnosis, addressing the underlying condition, and excising infected tissue through surgical intervention [11]. Early aggressive intervention with antifungal therapy, such as LAmB, combined with surgery is critical for halting progression and reducing mortality [4]. Additionally, delayed-release tablets or intravenous posaconazole and isavuconazole are recommended second-line treatments [4]. After an urgent nephrectomy, our patient received LAmB. However, fluid accumulation persisted within the liver and resected kidney space. Mucormycosis can disseminate through fascia, potentially presenting with fistulas [12]. The development of a bronchopleural fistula is a risky complication of pulmonary mucormycosis and *Klebsiella* pneumonia, which was later isolated in our patient [12,13]. In this patient, a follow-up CT with contrast confirmed the presence of a bronchopleural fistula, which required surgical intervention. Fluid accumulation ceased post-operatively. The prognosis for RM is poor, with nearly a 100 % mortality in patients experiencing bilateral renal involvement and acute kidney failure [8]. Unilateral renal involvement, urgent surgical intervention, along with timely and appropriate antifungal treatment are essential factors that alleviated the high mortality risk in our patient.

## 3 Conclusion

Developing awareness of RM plays a vital role in identifying and preventing complications and fatalities. Hence, we recommend extra caution in recognizing and promptly treating RM while paying close attention to the development of bronchopleural fistulas in the presence of persistent fluid accumulation post-surgery. This particular diagnosis along with its complications are frequently overlooked.

## Funding source

None.

## Consent for publication

Written informed consent was obtained from the patient for publication of this case report and accompanying images. A copy of the written consent is available for review on request.

## Ethical approval statement

Given the nature of the article, a case report, no ethical approval was required.

## Data availability statement

No data was used for the research described in the article.

## CRediT authorship contribution statement

**Ali Jawad:** Writing – review & editing, Writing – original draft. **Zein Alabdin Hannouneh:** Writing – review & editing, Writing – original draft. **Hadi Salame:** Writing – review & editing, Writing – original draft. **Hussein Taher:** Writing – review & editing, Writing – original draft. **Banan Alkharat:** Writing – review & editing, Supervision.

## Declaration of competing interest

The authors declare that they have no known competing financial interests or personal relationships that could have appeared to influence the work reported in this paper.
